# Disseminated Histoplasmosis in an Immunocompetent Host Presenting as Pancytopenia with Bilateral Adrenal Masses

**DOI:** 10.4274/tjh.2014.0084

**Published:** 2015-05-08

**Authors:** Smeeta Gajendra, Bhawna Jha, Tushar Sahni, Shalini Goel, Vimarsh Raina, Ritesh Sachdev

**Affiliations:** 1 Medanta The Medicity Hospital, Clinic of Pathology and Laboratory Medicine, Haryana, India

**Keywords:** Histoplasma, Pancytopenia, Adrenal masses

A 44-year-old male presented with fever, progressive weight loss, and anorexia for 6 months. The laboratory results showed deranged renal function tests. Serum adrenocorticotropic hormone was high at 252 pg/mL (normal limit: <46 pg/mL), suggestive of primary adrenal insufficiency. Serum free light chains were elevated, kappa at 87.97 mg/L (reference range: 3.30-19.40 mg/L) and lambda at 91.77 mg/L (reference range: 5.71-26.30). Ultrasonography of the abdomen showed hepatosplenomegaly with space-occupying lesions in bilateral suprarenal regions, while endoscopy ultrasound-guided fine-needle aspiration showed necrotizing granulomatous inflammation. Work-up for tuberculosis and human immunodeficiency virus was negative. The hematological parameters showed pancytopenia. The bone marrow aspiration revealed round to oval organisms with crescent-shaped eccentric nuclei both extracellularly and intracellularly, inside the macrophages and osteoclastic giant cells (Figure 1A). Bone marrow biopsy showed the presence of intracellular and extracellular oval capsulated globose organisms spread throughout the marrow spaces (Figure 1B). Periodic acid-Schiff (PAS) staining showed these organisms as bright eosinophilic structures with clear halos around them (Figure 1C). Gomori methenamine silver (GMS) staining showed clusters of fungal yeasts, morphologically compatible with Histoplasma capsulatum (Figure 1D). The patient was started with intravenous amphotericin B followed by oral itraconazole. His condition improved with recovery of counts and improvement of renal function; he is currently doing well. Informed consent was obtained.

Histoplasmosis is a fungal infectious disease caused by inhalation of spores of Histoplasma capsulatum. It may present as a self-limiting form or progressive disseminated disease. Disseminated histoplasmosis may affect almost all systems, including the reticuloendothelial system, lungs, gastrointestinal tract, renal tract, central nervous system, visual system, bone marrow, and adrenal glands [1]. Histoplasmosis presenting as a bilateral adrenal mass in an immunocompetent patient is rare. A high index of suspicion is required for the diagnosis of disseminated histoplasmosis in a patient with unexplained fever, as it may mimic other chronic illnesses or a neoplasm. The differential diagnoses that should be considered are tuberculosis, sarcoidosis, adrenal hemorrhage, metastatic carcinoma, and lymphoma. Despite extensive imaging, positron emission tomography scanning, and fine-needle aspiration biopsy, a definite diagnosis may not be reached [2]. Bone marrow examination is a useful diagnostic test to establish a diagnosis of disseminated histoplasmosis. In our case, a middle-aged immunocompetent patient presented with nonspecific symptoms and bilateral adrenal mass with insufficiency, the diagnosis of which was only possible with bone marrow examination.

**Conflict of Interest Statement**

The authors of this paper have no conflicts of interest, including specific financial interests, relationships, and/or affiliations relevant to the subject matter or materials included.

## Figures and Tables

**Figure 1:**
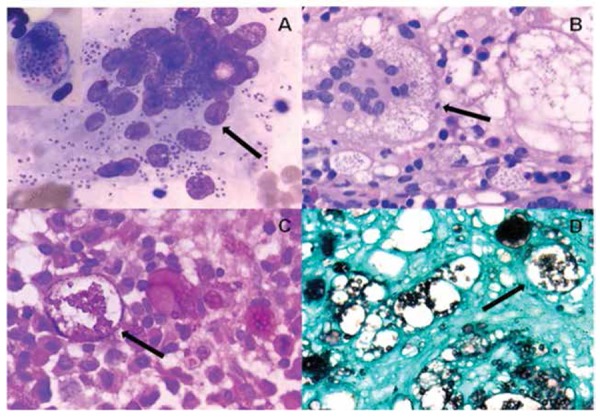
A) Bone marrow aspirate showing numerous Histoplasma capsulatum inside the osteoclastic giant cell and macrophage (inset) (Giemsa, 100^x^), B) bone marrow biopsy showing Histoplasma (H&E, 100^x^), C) PAS staining showed yeast-like cells with bright eosinophilic structures, D) GMS staining showed clusters of fungal yeasts.
